# Effortful and effortless training of executive functions improve brain multiple demand system activities differently: an activation likelihood estimation meta-analysis of functional neuroimaging studies

**DOI:** 10.3389/fnins.2023.1243409

**Published:** 2023-11-14

**Authors:** Chan Tang, Ting Huang, Jipeng Huang, Nuo Xu, Hui Lyu, Yuan Wang, Yifei Cao

**Affiliations:** ^1^School of Psychology, Northeast Normal University, Changchun, China; ^2^School of Psychology, Jilin Provincial Key Laboratory of Cognitive Neuroscience and Brain Development, Northeast Normal University, Changchun, China; ^3^State Key Laboratory for Cognitive Neuroscience and Learning, Faculty of Psychology, Beijing Normal University, Beijing, China; ^4^School of Humanities and Social Sciences, Beijing Institute of Technology, Beijing, China; ^5^Zurich Center for Neuroscience, University of Zurich and ETH Zurich, Zurich, Switzerland

**Keywords:** effortful training, effortless training, executive functions, multiple demand system, fMRI, meta-analysis

## Abstract

Both effortful and effortless training have been shown to be effective in enhancing individuals' executive functions. Effortful training improves domain-specific EFs, while effortless training improves domain-general EFs. Furthermore, effortful training has significantly higher training effects on EFs than effortless training. The neural mechanism underlying these different effects remained unclear. The present study conducted meta-analysis on neuroimaging studies to explore the changes of brain activations induced by effortful and effortless training. The results showed that effortful training induced greater activation in superior frontal gyrus, while effortless training induced greater activation in middle frontal gyrus, precuneus and cuneus. The brain regions of MD system enhanced by effortful training were more associated with core cognitive functions underlying EFs, while those enhanced by effortless training were more correlated with language functions. In addition, the significant clusters induced by effortful training had more overlaps with the MD system than effortless training. These results provided us with possibility to discuss the different behavioral results brought by effortful and effortless training.

## 1 Introduction

Executive functions (EFs) refer to human's ability to form goals, make and implement plans effectively (Lezak, 1982), which has three basic components: updating, inhibition, and shifting (Diamond, 2013). Updating refers to the ability of keeping and manipulating information, inhibition refers the ability to ignore distraction and inhibit automatic responses, and shifting refers to ability of switching flexibly between different rules and mental processes (Miyake et al., 2000; Diamond and Lee, 2011). EFs are generally considered to be the basis of various cognitive abilities which play a critical role in human cognitive development (Espy, 2004; Diamond, 2013), and the impairment of EFs are frequently observed in populations with developmental disorders including attention deficit hyperactivity disorder (ADHD), autism spectrum disorder (ASD), and others (Weyandt, 2009; Barendse et al., 2013; Craig et al., 2016). Therefore, researchers have been investigating how to enhance individuals' EFs using various effective interventions (Klingberg et al., 2005; Karbach and Kray, 2009; Johann and Karbach, 2020).

Training programs developed could be divided into two groups: effortful and effortless training. Effortful training, designed to make trainees engage their cognitive resource in order to achieve the certain goal (Kahneman, 1973), was the mainstream training method. Some of these programs train participants with cognitive tasks that invoke specific EFs su bcomponent such as N-back task for working memory (Buschkuehl et al., 2014; Heinzel et al., 2016), stop-signal task for inhibitory control (Berkman et al., 2014; Wang et al., 2020), and switching task for cognitive flexibility (Espinet et al., 2013; van Bers et al., 2020). Other effortful training programs train with software and game-based computer tasks, for example, Cogmed and Lumosity (van der Donk et al., 2015; Steyvers et al., 2019; Kelly et al., 2020; Steyvers and Schafer, 2020). For effortless training, this emerging trend of method hypothesize that cognition could be improved in programs that engage minimal cognitive effort (Moreau and Conway, 2014; Tang et al., 2022). In this kind of training approaches, participants were trained with mindfulness practice (Van de Weijer-Bergsma et al., 2012; Nien et al., 2020), physical exercise (Krafft et al., 2014; Hsu et al., 2018; Kleinloog et al., 2019), and musical training (Moreno et al., 2011; Guo et al., 2021).

Training effects brought by training programs could be divided into near and far transfer effects: the former one refers to the improvement of performance on the tasks measured the same or similar cognitive abilities, and the latter one refers to the improvement of performance on tasks measuring different cognitive domains (Barnett and Ceci, 2002; Sala et al., 2019). Previous training and meta-analysis studies have indicated the different effects of effortful and effortless training on individuals' EFs. Since effortful programs were designed to improve specific EFs domains, performance in the same or similar cognitive task could be enhanced in these programs (Cao, 2019; Takacs and Kassai, 2019; Scionti et al., 2020) but less enhancement in tasks measuring different EFs subdomain from the trained task (Kassai et al., 2019; Sala and Gobet, 2019; Cao et al., 2020). On the other hand, effortless training programs aimed at no specific EFs domain, and training effects brought by this kind of programs could be viewed as far-transfer effects. Therefore, the training effects of effortless training were significantly lower than effortful training (Takacs and Kassai, 2019) but showing no difference between the gains in different EFs subdomains (Chen et al., 2020). The difference between the training effects indicates that the two training approaches improve EFs from distinct mechanisms, effortful training influence the domain-specific process of trained subdomain, while effortless training improves relatively domain-general factors of EFs.

Distinct training effects of the two training approaches might origin from different brain activity elicited. However, the neural mechanism underlying the different training effects on behavior performance remains unclear. Previous meta-analysis on neuroimaging studies revealed that effortful training could induce greater activations in the medial frontal gyrus, inferior parietal louble, and precuneus (Li et al., 2015; Vartanian et al., 2022). While for effortless training, Tang et al. (2022) raised hypothesis that attention and self-control (including task performance on EFs) could be enhanced by effortless training through the ACC-PCC-striatum (APS) circuit. Meta-analytic evidence still lacks to examine the neural mechanism underlying the effects of effortless training on EFs. The multiple demand (MD) system are the core brain regions that widely believed to closely related with human intelligence and EFs, since these regions activate under a variety of tasks with various demands, mainly composed of brain regions of the prefrontal lobe and parietal lobe (Duncan, 2010; Woolgar et al., 2018). Furthermore, past studies have also found that both effortful and effortless training could induce changes of brain activations within the MD system (Li et al., 2015; Mothersill and Donohoe, 2019; Yu et al., 2021; Vartanian et al., 2022). Therefore, we speculate that besides the different behavioral effects, effortful and effortless training could also induce different brain activity changes within MD system.

In the present study, we conducted a neuroimaging meta-analysis using activation likelihood estimation (ALE) to examine the neural basis of effortful and effortless training on EFs (Eickhoff et al., 2009, 2012). Next, we conducted contrast and conjunction analysis to reveal the distinct and common brain regions influenced by the two training approaches.

## 2 Methods

### 2.1 Data sources

Literature search was conducted through Web of Science and Scopus. For the search strings, we used keywords that represents EFs, different training approaches, and fMRI. We searched for the literature published after January of 2000 and until the search date. Furthermore, reference lists of the included articles were manually searched for not missing relevant articles for the topic. In total, 62 studies containing 81 experiments were included.

### 2.2 Inclusion criteria and study selection

**Figure 1:**
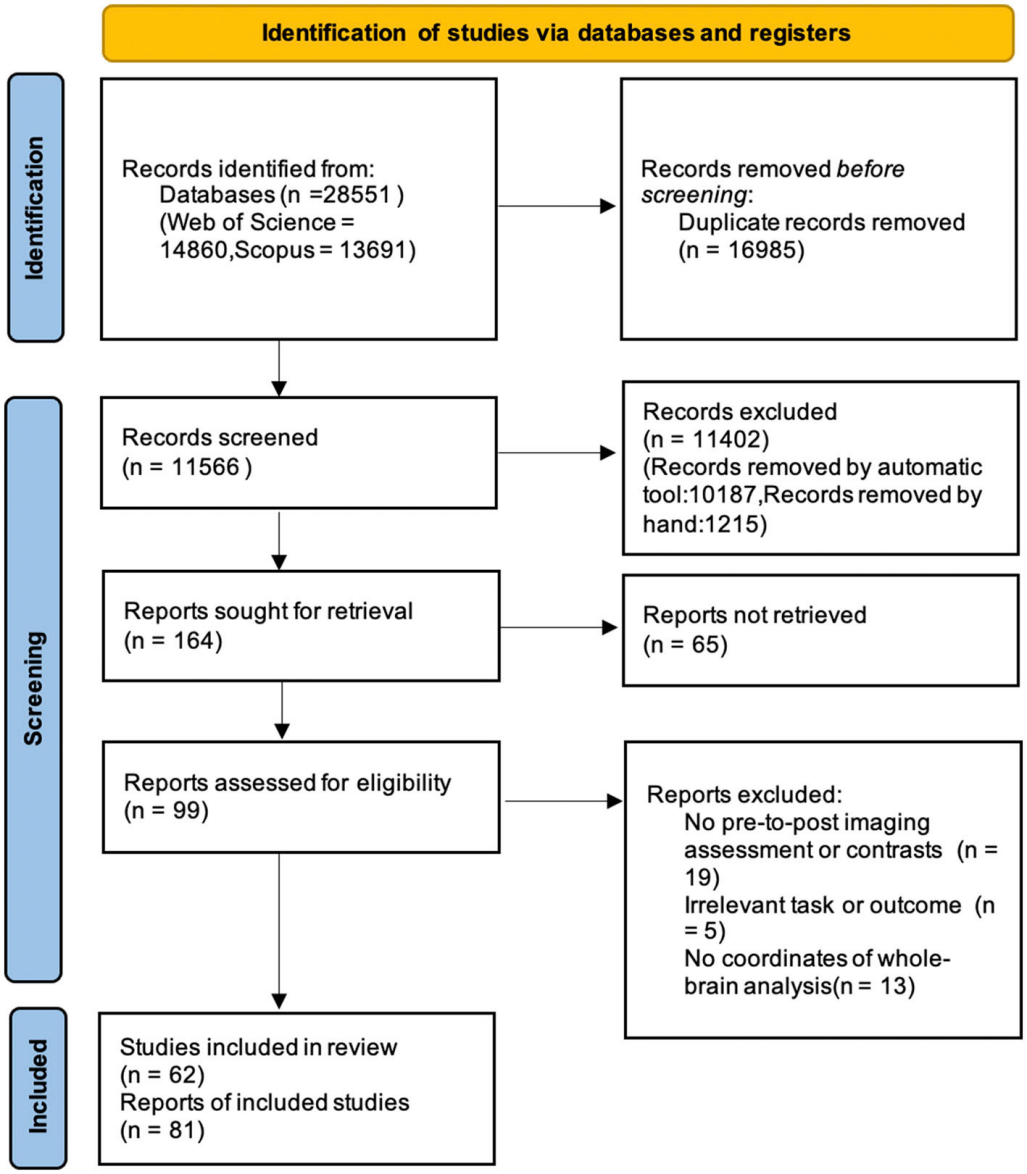
Flowchart for study including and screening.

The screen for relevant studies was conducted corresponding to PICOS-principles whose full descriptions are participants (P), intervention (I), comparisons (C), outcomes (O), and study design (S). The initial search revealed 28,551 articles. We incorporated articles when they met the following criteria: (1) studies employed fMRI technique to explore human brain activity related to the review topic were included, (2) studies adopted a pretest-training-posttest pattern were included while research only did one set of fMRI scanning were excluded, (3) studies reporting activation and deactivation data from subtractions between posttest and pretest or baseline conditions were included, and (4) studies reported results in detailed coordinates of whole-brain analysis in standard reference space and with significant effect were included. Studies without sufficient data to perform ALE analysis after contacting to the authors were also excluded. Due to the large number of identified records from dataset, we used software ASReview Lab v1.2.1 (Van De Schoot et al., 2021) for aiding the literature screening process. Ninety-nine studies were remained for successfully meeting all the criteria and obtaining full-text. The full-text assessment was done by two independent authors, which results 59 eligible studies with 69 experiments. A flow chart illustrating the detailed literature searching and study selection process can be viewed in Figure 1.

### 2.3 Coding of variables

The extraction of the relevant data was done with the following details: (1) name of the lead author; (2) publication year; (3) population characteristics (e.g., health type, age, and male percentage), training characteristics (e.g., participants number, training duration and frequency, and control condition); (4) cognitive task paradigms employed to access the effect of training and the type of cognition it tested; and (5) training type, effortful training programs were operationalized as programs that specifically targeted one or more components of EF as proposed by Miyake et al. (2000) namely, working memory, inhibitory control, and cognitive flexibility, while effortless training programs were programs those engage minimal mental effort and involve effortless practices or experiences, such as nature exposure and flow experience (e.g., mindfulness, physical activity, and musical training).

### 2.4 ALE analysis

Meta-analysis were conducted based on the ALE method (Laird et al., 2005) using GingerALE 3.0.2 (https://www.brainmap.org/ale/). The algorithm aims at determining the consistent locations of brain activation in studies using similar experimental conditions. In ALE, activation focis are treated as centers for the probability distributions capturing the spatial uncertainty associated with each focus. The probabilities of all foci reported in a given experiment were then calculated to form the voxel-wise ALE score maps using an automatically determined full-width half-maximum (FWHM) value (Eickhoff et al., 2009), which is calculated by the number of subjects in each experiment. The size of the FWHM of the Gaussian kernel was adjusted for the expected between-subject and between-template variability to model spatial uncertainty (Turkeltaub et al., 2011). Next, in order to test whether the convergence was reliable, ALE maps were compared to null-distributions acquired from independent studies' ALE values. The *p*-value was given by the proportion of equal or higher values under the null-distribution. To correct for multiple comparisons, we applied stringent threshold algorithms of family-wise error rate (FWE) *p* < 0.05 (1,000 permutations for uncorrected *p* < 0.001) to reveal the training-induced effects (Eickhoff et al., 2012).

Statistical comparisons between two ALE maps were conducted also based on GingerALE, using conjunction and contrast analysis (Nichols et al., 2005; Eickhoff et al., 2011; Rottschy et al., 2012). These analysis uncovered the similarity and differences in tr aining effects between effortful and effortless training. We applied a threshold of FDR pN < 0.01 (10,000 permutations), cluster size > 200 mm^3^ to the conjunction, and contrast analysis. Finally, the GingerALE software identified the brain locations of significant clusters detected in the meta-analysis.

### 2.5 MD system ROI

**Figure 2:**
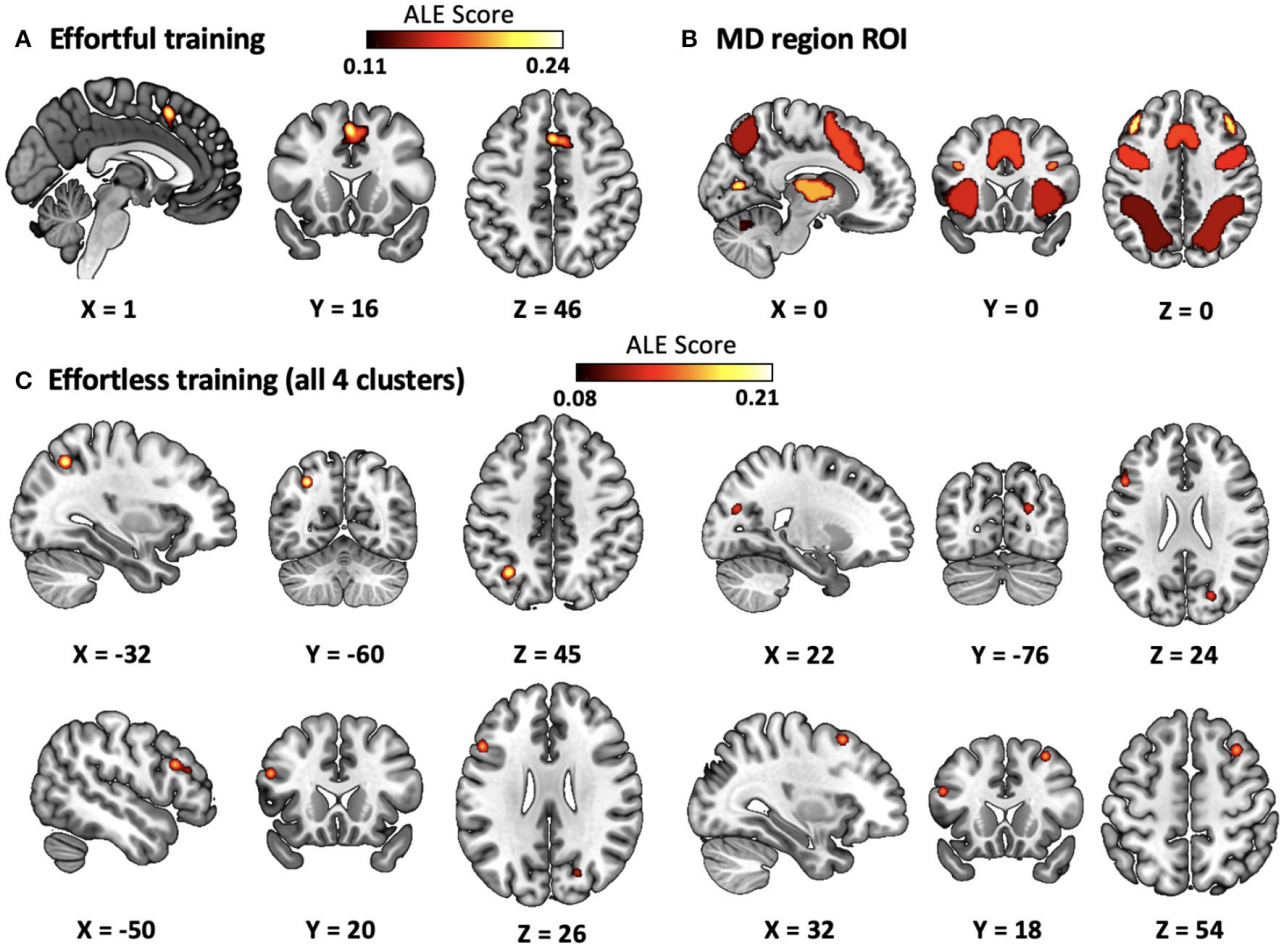
Results for significant clusters revealed by individual and contrast analysis. **(A)** Meta-analysis of effortful training. **(B)** ROI of MD system. **(C)** Meta-analysis of effortless training. ALE, activation likelihood estimation.

To examine the overlap between the significant clusters and human MD system, we selected the MD system as ROI (Figure 2B). The MD network was based on data from Fedorenko et al. (2013), selecting frontoparietal regions responsive to cognitive demands across seven diverse tasks (http://imaging.mrc-cbu.cam.ac.uk/imaging/MDsystem).

### 2.6 Meta-analytic functional decoding

Using the Neurosynth Image Decoder, we decoded the functional characteristics of meta-analysis network of the sub-group meta-analysis result maps of effortful and effortless training. The decoder calculates the similarity between any meta-analysis network and other meta-analytical maps related to certain terms by computing Pearson's correlation coefficients across all voxels (Bellucci et al., 2019). We selected the top 10 terms associated with each training approaches to represent the most related cognitive functions.

## 3 Results

### 3.1 Characteristics of included studies

There were total 408 foci of activation information within 59 studies, with a total of 1,167 subjects (*M*_age_ = 34.31, SD_age_ = 20.24): 782 healthy people and 385 patients. Forty four studies trained participants with effortful training approaches, while the remaining fifteen studies used effortless training programs. For the subcomponent of EFs measured, 31 studies measured working memory, 26 studies measured inhibitory control, and three studies measured cognitive flexibility. Detailed study characteristic were elaborated in Supplementary Table 1.

There were total 408 foci of activation information within 61 studies, with a total of 1,167 subjects (*M*_age_=34.31, SD_age_ = 20.24): 782 healthy people and 385 patients. Thirty two studies trained participants with effortful training approaches (with 71.9% of them measured working memory, 25% of them measured inhibition, and 3.1% of them measured flexibility), while the remaining twenty nine studies used effortless training programs (with 28% of them measured working memory, 68% of them measured inhibition, and 4% measured flexibility). For the age groups of studies included, 17 studies (28.6%) trained children (age range: 0–18), 33 studies (51.7%) trained adults (age range: 19–64), and 12 studies (19.7%) trained the elderly (age range: above 65). Detailed study characteristic were elaborated in Supplementary Table 1.

### 3.2 Individual meta-analysis

First, we examined the overall meta-analysis result of the fifty-nine studies that investigated the effect of cognitive training on individuals' executive functions. Results showed that convergence occurred in the multiple-demand network, with the activation peaks appeared at superior frontal gyrus (extending to cingulate gyrus and medial frontal gyrus, see Table 1). Additionally, it should be aware that there are two types of scopes for the superior frontal gyrus. In the first opinion, the superior frontal gyrus is the gyrus located on the frontal lobe's superolateral surface (and does not extend to the medial surface of the frontal lobe), while in the second opinion, the superior frontal gyrus is the superior part of the interhemispheric (medial) surface of the frontal lobe (Damasio and Woods, 1995; Tamraz et al., 2004; Drake et al., 2009). According to the location of the overall cluster given by GingerALE, this software defined superior frontal gyrus followed the second opinion.

**Table 1:** Significant clusters revealed by individual meta-analysis.

**Cluster**	**Coordinates**			***Z*-value**	**Cluster size**	**Brain region**
	*x*	*y*	*z*			
**Overall**						
1	10	10	42	5.17	3216 mm^3^	Superior frontal gyrus (39.3%)
						Medial frontal gyrus (31.8%)
						Cingulate gyrus (28.9%)
**Effortful**						
1	1	16	46	4.13	2504 mm^3^	Superior frontal gyrus (43.2%)
						Medial frontal gyrus (33.1%)
						Cingulate gyrus (23.7%)
**Effortless**						
1	−32	−60	46	5.62	1064 mm^3^	Precuneus (43.9%)
						Inferior parietal lobule (24.4%)
						Superior parietal lobule (17.1%)
						Angular Gyrus (14.6%)
2	−50	20	26	5.00	984 mm^3^	Middle frontal gyrus (60.5%)
						Inferior Frontal Gyrus (39.5%)
3	22	−76	24	4.26	712 mm^3^	Cuneus (72.1%)
						Precuneus (27.9%)
4	32	18	54	4.93	664 mm^3^	Middle frontal gyrus (46.7%)
						Superior frontal gyrus (30%)
						Subgyral (23.3%)

Next, we conducted individual meta-analysis separately on studies using effortful and effortless training approaches. For efffortful training, the ALE meta-analysis of all foci (Table 1 and Figure 2A) revealed one significant cluster also located in the superior frontal gyrus (extending to medial frontal gurys and cingulate) with 46.2% voxels located within anterior cingulate cortex (ACC). The ALE meta-analysis of all foci reported in effortless dataset revealed four significant clusters (Table 2 and Figure 2C) including left precuneus (extending to inferior and superior parietal lobule, and angular gyrus), left middle frontal gyrus (extending to inferior frontal gyrus), right cuneus (extending to precuneus), and right middle frontal gyrus (extending to superior frontal gyrus).

**Table 2:** Contrast analysis results of effortless and effortful training.

**Cluster**	**Coordinates**			***Z*-value**	**Cluster size**	**Brain region**
	*x*	*y*	*z*			
**Effortful < effortless**						
1				−36	−62	40	2.86	408 mm^3^	Precuneus (56.3%)
	Angular gyrus (31.3%)							
	Inferior parietal lobule (12.5%)							
2	−48	26	24	2.55	248 mm^3^	Middle frontal gyrus (81.8%)
						Inferior frontal gyrus (18.2%)

Furthermore, in order to examine the extent of how effortful and effortless training improve brain activity in MD regions, we calculated the number of overlapping voxels between the significant clusters and MD region. The results revealed that 56% of voxels in significant cluster induced by effortless training locates within MD regions and 79% of voxels in significant cluster induced by effortful training locates within MD regions. Moreover, the significant clusters induced by effortful and effortless training overlapped with different parts of the MD system. Clusters in effortless training mainly overlapped with the inferior frontal sulcus, prefrontal cortex of MD system, while cluster in effortful training mainly overlapped with the pre-SMA and ACC part of the MD system.

### 3.3 Contrast and conjunction analysis

The contrast analysis between effortless and effortful training revealed two significant clusters locating in the precuneus (extending to angular gyrus and inferior parietal lobule) and middle frontal gyrus (extending to inferior frontal gyrus). No significant cluster was found in the conjuction analysis between the two training approaches. Therefore, the conjunction results indicated that though both training approaches improved the MD system, they enhanced different parts from each other.

### 3.4 Meta-analytica functional decoding

**Figure 3:**
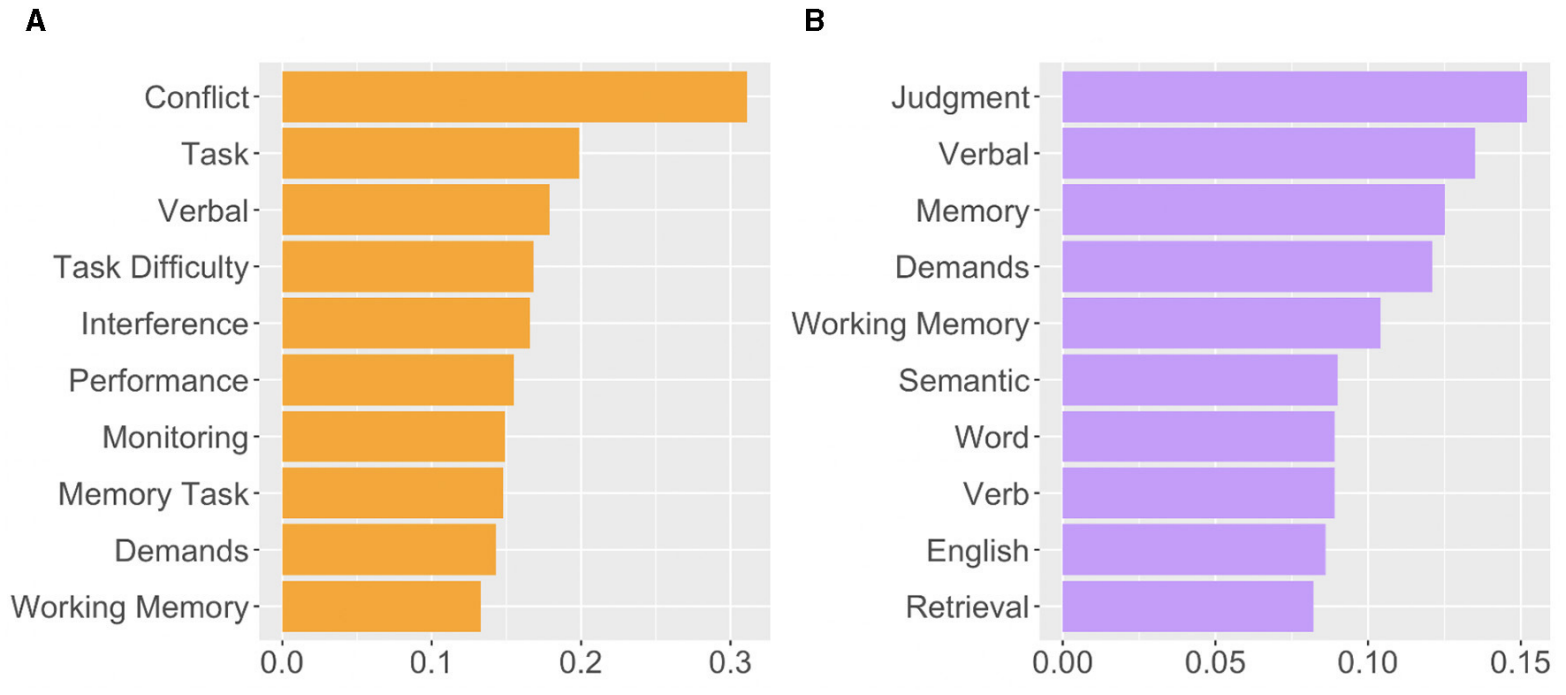
Results of meta-analytical functional decoding of **(A)** effortful training and **(B)** effortless training. The *y*-axis represents the terms associated with each training types, and *x*-axis represents the similarity (Pearson's correlation).

The Neurosynth Image Decoder indicated that the meta-analytical map of effortful training is primarily associated with terms representing conflict monitoring and task demand (Figure 3A), while effortless is more associated with terms representing judgement and language functions (Figure 3B).

## 4 Discussions

The current study has shown that both effortful and effortless training elicit significant changes of brain activation within the MD system during EFs tasks. However, there are differences exist between the effects of these two training approches on MD system. First, significant cluster induced by effortful training located in superior frontal gyrus, while those induced by effortless training located in precuneus, middle frontal gyrus, and cuneus. Second, effortful training induced most brain activation changes within the MD system, while only half of the effortless training induced clusters located within MD systems. These results contributed to comprehending different behavioral effects brought by these two training approaches.

For effortful training, the significant cluster located in the superior frontal gyrus has been shown to be cruicial for human EFs and cognitive control (Jobson et al., 2021; Friedman and Robbins, 2022). More importantly, ACC serves the cognitive control process by involving in multiple specific cognitive progress including pre-response conflict, decision uncertainty, response error, and negative feedback (Ridderinkhof et al., 2004). In addition, the superior frontal gyrus is the core brain region in MD system that support human general intelligence (Duncan, 2010). Therefore, the increased EFs of individuals brought by effortful training could be explained by the enhancement of the core brain region that supports human high-level cognition.

On the other hand, though effortless training also induced enhancement of MD system activities, the enhanced regions were different from effortful training and thus brought different behavioral results. First, the significant clusters induced by effortless training overlaps 23% less than effortful training, and this indicated that MD systems were more representative in effortful training than effortless training. Second, according to the hierarchical clustering of MD systems (Camilleri et al., 2018), the MD system could be divided into three cliques: “sub-cortical sub-group,” the “organizers,” and the “workers.” Specifically, regions in the “organizer” clique are more associated with planning and monitoring, while regions in the “worker” clique act like a heterogeneous set of workers dynamically recruited based on task demands. In the present study, the significant cluster induced by effortful training located within “the organizers” clique of the MD system, while those nduced by efforless training located within “The Workers” clique. Furthermore, according to the meta-analytical decoding results in the present study (see Figure 3), the meta-analytical result map of effortful training is more correlated with the terms associated with core cognitive functions supporting EFs, while the map of effortless training is mainly correlated with terms associated with judgement and semantic functions. Taking these finding together, we could reach a conclusion that effortful training is strongly correlated with the core brain regions that support human EFs and intelligence, and this is consistent with the phenomenon that effortful training could induce larger training effects on the behavioral performance of human EFs (Takacs and Kassai, 2019).

In conclusion, the different brain clusters found in the present meta-analysis revealed the potential neural mechanisms for effortful and effortless training on human EFs. However, some limitations still exist. First, due to limited number of studies, we could not further conduct aubgroup analysis to examine the effect of different sub-types of training on different subdomains of EFs, and the different training effects on different population characteristics (e.g., age and health type). Since there might be different training effects between young and old participants (Braver et al., 2009), healthy and unhealthy participants (Cao et al., 2020), the present results including different populations might caused by mixed results. Second, present results from ALE meta-analysis cannot illustrate causal relationship between the neural and behavioral indices, and further neuroimaging studies could be conducted to examine the possible causal relationships.

## Data availability statement

The datasets presented in this study can be found in online repositories. The names of the repository/repositories and accession number(s) can be found at: https://github.com/YifeiCAO/Meta-analysis-data.

## Author contributions

Conceptualization and writing—review and editing: YC and YW. Methodology: TH, JH, NX, and HL. Investigation: CT, TH, YC, and YW. Supervision: YC. Writing—original draft: CT, TH, and YC. All authors contributed to the article and approved the submitted version.
