# Supplementary material for: Effortful and effortless training of executive functions improve brain multiple demand system activities differently: an activation likelihood estimation meta-analysis of functional neuroimaging studies
Source: Front Neurosci. 2023 Nov 14;17:1243409. doi: 10.3389/fnins.2023.1243409 (PMC10682784; doi:10.3389/fnins.2023.1243409)
Supplement: Supplementary file 1 [file Table_1.pdf]

Table 1 Information about included papers

Study	N	Contrast	Paradigm	Domain	Training type	Duration (mins)	mean age	health type	control	Male percent
Adnan(2017)	11	post>pre	face/scene delayed matching task	WM/inhibition	Effortful	1200	65.9	healthy	+	0.27
Allen(2012)	30	post>pre	Stroop task	inhibition	Efforless	720	NA	healthy	+	NA
Ando(2009)	4	post>pre	visuospatial WM task	WM	Effortful	NA	NA	healthy	-	1.00
Bachmann(2018)	21	post>pre	n-back	WM	Efforless	1200	40	ADHD	+	0.38
Brehmer(2011)	12	both	spatial delayed-matching task	high-level WM	Effortful	500	63.7	healthy	+	0.50
Berkman (2014)	30	both	stop-signal	inhibition	Effortful	120	21.63	healthy	-	0.45
Bor (2011)	8	post>pre	n-back	WM	Effortful	1680	30.5	schizophrenia	-	0.75
Brockmeyer (2016)	12	post>pre	target-detection	flexibility	Effortful	945	22.82	anorexia nervosa	-	NA
Brockmeyer (2016)	12	post>pre	target-detection	inhibition	Effortful	945	22.82	anorexia nervosa	-	NA
Buschkuehl(2014)	27	post>pre	n-back	WM	Effortful	140	22.3	healthy	+	0.63
Chavan (2015)	18	post<pre	Go/NoGo	inhibition	Effortful	840	25.1	healthy	-	0.44
Chavan (2015)	18	post<pre	n-back	WM	Effortful	840	25.1	healthy	-	0.44
Cho(2014)	12	post>pre	n-back	WM	Efforless	28	60.58	subjective memory complaints	NA	0.00
Clark(2017)	25	post>pre	dual n-back	WM	Effortful	600	30.68	healthy	+	0.44
Conklin(2015)	34	post<pre	spatial WM task	WM	Effortful	750-1125	12.21	cognitive deficits	-	0.53
Davis(2011)	10	both	antisaccade	inhibition	Efforless	3640	9.6	overweight	-	0.60

DeVito (2012)	12	post<pre	stroop	inhibition	Effortful	NA	37.2	substance use disorder	-	0.58
DeVito(2017)	26	post<pre	stroop task	inhibition	Efforless	600	40.27	cocaine-use disorder	-	NA
DeVito(2018)	10	post<pre	color-word stroop	inhibition	Efforless	NA	NA	cocaine-use disorder	-	NA
Dong(2016)	18	post<pre	digit/letter memory span visuospatial n-back task	WM	Efforful	1800	21.38	healthy	-	0.94
Emch(2019)	30	post>pre	verbal WM task	WM	Effortful	960	55.8	healthy	+	0.50
Filippi (2012)	10	post>pre	stroop	inhibition	Effortful	2160	44.8	multiple sclerosis	-	0.00
Gunning (2021)	34	post>pre	stroop	inhibition	Effortful	400	61.6	depression	NA	NA
Guo(2020)	27	post<pre	visual WM task	WM	Efforless	960	73.3	healthy	-	0.22
Harl´e(2019)	20	both	stop-signal	inhibition	Efforless	null	31.95	PTSD	NA	1.00
Heinzel(2016)	15	both	n-back+sternberg	WM	Effortful	540	66.07	healthy	-	0.40
Hiroyasu(2015)	5	post>pre	reading span task	WM	Effortful	NA	22.3	healthy	-	0.82
Hoekzema(2010)	10	post>pre	Go/NoGo	inhibition	Efforless	450	11.1	ADHD	+	0.80
Hsu(2017)	10	post<pre	Eriksen Flanker task	inhibition	Efforless	4320	71.5	vascular cognitive impairment	-	0.57
Huyser (2011)	25	post>pre	flanker	inhibition	Efforless	NA	13.95	OCD	-	0.36
Huyser (2010)	25	post>pre	tower of London	planning	Efforless	NA	13.95	OCD	-	0.36
Hwang(2018)	25	both	stroop	inhibition	Efforless	450	46.86	chronic insomnia disorder	-	0.24
Jolles(2010)	15	both	WM task	WM	Effortful	400	22.04	healthy	-	0.47

Jolles(2012)	11	both	WM task	WM	Effortful	375	12.35	healthy	-	0.40
Kang (2017)	16	post<pre	switch	flexibility	Effortful	240	22.1	healthy	-	0.38
Kelly (2019)	9	post>pre	n-back	WM	Effortful	900	8.1	low birth weight	-	0.33
Kim(2017)	14	post>pre	multisource interference task	inhibition	Effortful	960	71.57	healthy	-	0.07
Krafft(2014)	24	post<pre	antisaccade/flanker	inhibition	Efforless	5520	9.7	overweight	+	0.29
Martinsen(2018)	19	post>pre	word-color stroop	inhibition	Efforless	1800	49.6	fibromyalgia	-	0.00
Meusel(2013)	23	post>pre	n-back	WM	Effortful	1800	48.4	mood disorders	-	0.20
Nash(2017)	10	both	Go/NoGo	inhibition	Effortful	1080	10.3	fetal alcohol spectrum disorder	-	0.50
Necka(2021)	23	both	n-back	WM	Effortful	300-350	27.20	healthy	-	0.33
Necka(2021)	23	both	n-back	inhibition	Effortful	300-350	27.20	healthy	-	0.33
Newman(2016)	14	post>pre	mental rotation task	WM	Effortful	150	8.3	healthy	-	0.54
Nishiguchi(2015)	24	post>pre	n-back	WM	Efforless	1080	73	healthy	-	0.54
Nombela(2011)	5	both	stroop	inhibition	Efforless	NA	60.1	parkinson	-	0.60
Olesen(2004)	3	post>pre	VSWM/backwards digit span task/letter span task	WM	Effortful	500	22	healthy	-	1.00
Olesen(2004)	8	post>pre	VSWM/backwards digit span task/letter span task	WM	Effortful	360	29.3	healthy	-	0.75
Osaka(2012)	25	post>pre	reading span task	WM	Effortful	60	68.08	healthy	-	0.68
Ott(2020)	26	both	spatial n-bask WM task	WM	Effortful	2400	36	bipolar disorder	-	0.23
Padilla(2019)	25	post<pre	n-back	WM	Effortful	200	22.77	healthy	-	0.36
Padilla(2020)	25	post>pre	n-back	WM	Effortful	200	22.72	healthy	-	0.40

Pensel(2018)	23	both	flanker	inhibition	Efforless	6480	49	healthy	-	1.00
Ramsay (2016)	14	post>pre	n-back	WM	Effortful	960	42.93	schizophrenia	-	NA
Salminen(2016)	18	both	dual n-back	WM	Effortful	480	24.4	healthy	+	0.33
Siniatchkin (2012)	17	post>pre	Go/NoGo	inhibition	Efforless	6000	9.3	ADHD	-	0.82
Straten (2017)	15	post>pre	tower of London	planning	Efforless	NA	14.2	OCD	-	0.41
Thomaes(2012)	16	both	stroop task	inhibition	Efforless	NA	33.5	PTSD	-	0.00
Thorsen (2020)	31	both	stop-signal	inhibition	Efforless	4	0.39	OCD	-	0.39
Wu (2018)	16	post>pre	switch	flexibility	Efforless	2160	64.9	healthy	-	0.19
Wykes(2002)	6	post>pre	n-back	WM	Efforless	2400	35	schizophrenia	+	1.00
Zhu(2021)	14	post>pre	n-back	WM	Efforless	NA	11.81	healthy	+	0.57

Abbr: N: number of participants within training group;WM: working memory; CBT: cognitive behavior therapy; For control type, + represents active control, and – represents passive control.

REFERENCES

Adnan, A., Chen, A. J., Novakovic-Agopian, T., D'Esposito, M., and Turner, G. R. (2017). Brain changes following executive control training in older adults. *Neurorehabilitation and neural repair* 31, 910–922

Allen, M., Dietz, M., Blair, K. S., van Beek, M., Rees, G., Vestergaard-Poulsen, P., et al. (2012). Cognitive-affective neural plasticity following active-controlled mindfulness intervention. *Journal of Neuroscience* 32, 15601–15610

Ando, T., Momose, K., Tanaka, K., and Saito, K. (2009). Effects of task difficulty and training of visuospatial working memory task on brain activity. In *13th International Conference on Biomedical Engineering: ICBME 2008 3–6 December 2008 Singapore* (Springer), 657–660

Bachmann, K., Lam, A. P., Sörös, P., Kanat, M., Hoxhaj, E., Matthies, S., et al. (2018). Effects of mindfulness and psychoeducation on working memory in adult adhd: A randomised, controlled fmri study. *Behaviour research and therapy* 106, 47–56

Berkman, E. T., Kahn, L. E., and Merchant, J. S. (2014). Training-induced changes in inhibitory control network activity. *Journal of Neuroscience* 34, 149–157

Bor, J., Brunelin, J., d'Amato, T., Costes, N., Suaud-Chagny, M.-F., Saoud, M., et al. (2011). How can cognitive remediation therapy modulate brain activations in schizophrenia?: An fmri study. *Psychiatry Research: Neuroimaging* 192, 160–166

Brehmer, Y., Rieckmann, A., Bellander, M., Westerberg, H., Fischer, H., and Bäckman, L. (2011). Neural correlates of training-related working-memory gains in old age. *Neuroimage* 58, 1110–1120

Brockmeyer, T., Walther, S., Ingenerf, K., Wild, B., Hartmann, M., Weisbrod, M., et al. (2016). Brain effects of computer-assisted cognitive remediation therapy in anorexia nervosa: a pilot fmri study. *Psychiatry Research: Neuroimaging* 249, 52–56

Buschkuehl, M., Hernandez-Garcia, L., Jaeggi, S. M., Bernard, J. A., and Jonides, J. (2014). Neural effects of short-term training on working memory. *Cognitive, Affective, & Behavioral Neuroscience* 14, 147–160

Chavan, C. F., Mouthon, M., Draganski, B., Van Der Zwaag, W., and Spierer, L. (2015). Differential patterns of functional and structural plasticity within and between inferior frontal gyri support training-induced improvements in inhibitory control proficiency. *Human brain mapping* 36, 2527–2543

Cho, S.-Y., Jahng, G.-H., Rhee, H. Y., Park, S.-U., Jung, W.-S., Moon, S.-K., et al. (2014). An fmri study on the effects of jaw-tapping movement on memory function in elderly people with memory disturbances. *European Journal of Integrative Medicine* 6, 90–97

Clark, C. M., Lawlor-Savage, L., and Goghari, V. M. (2017). Functional brain activation associated with working memory training and transfer. *Behavioural Brain Research* 334, 34–49

Conklin, H. M., Ogg, R. J., Ashford, J. M., Scoggins, M. A., Zou, P., Clark, K. N., et al. (2015). Computerized cognitive training for amelioration of cognitive late effects among childhood cancer survivors: a randomized controlled trial. *Journal of clinical oncology* 33, 3894

Davis, C. L., Tomporowski, P. D., McDowell, J. E., Austin, B. P., Miller, P. H., Yanasak, N. E., et al. (2011). Exercise improves executive function and achievement and alters brain activation in overweight children: a randomized, controlled trial. *Health psychology* 30, 91

DeVito, E. E., Dong, G., Kober, H., Xu, J., Carroll, K. M., and Potenza, M. N. (2017). Functional neural changes following behavioral therapies and disulfiram for cocaine dependence. *Psychology of Addictive Behaviors* 31, 534

DeVito, E. E., Kober, H., Carroll, K. M., and Potenza, M. N. (2019). fmri stroop and behavioral treatment for cocaine-dependence: Preliminary findings in methadone-maintained individuals. *Addictive behaviors* 89, 10–14

DeVito, E. E., Worhunsky, P. D., Carroll, K. M., Rounsaville, B. J., Kober, H., and Potenza, M. N. (2012). A preliminary study of the neural effects of behavioral therapy for substance use disorders. *Drug and alcohol dependence* 122, 228–235

Dong, S., Wang, C., Xie, Y., Hu, Y., Weng, J., and Chen, F. (2016). The impact of abacus training on working memory and underlying neural correlates in young adults. *Neuroscience* 332, 181–190

Emch, M., Ripp, I., Wu, Q., Yakushev, I., and Koch, K. (2019). Neural and behavioral effects of an adaptive online verbal working memory training in healthy middle-aged adults. *Frontiers in aging neuroscience* 11, 300

Filippi, M., Riccitelli, G., Mattioli, F., Capra, R., Stampatori, C., Pagani, E., et al. (2012). Multiple sclerosis: effects of cognitive rehabilitation on structural and functional mr imaging measures—an explorative study. *Radiology* 262, 932–940

Gunning, F. M., Anguera, J. A., Victoria, L. W., and Areán, P. A. (2021). A digital intervention targeting cognitive control network dysfunction in middle age and older adults with major depression. *Translational psychiatry* 11, 269

Guo, X., Yamashita, M., Suzuki, M., Ohsawa, C., Asano, K., Abe, N., et al. (2021). Musical instrument training program improves verbal memory and neural efficiency in novice older adults. *Human Brain Mapping* 42, 1359–1375

Harlé, K. M., Spadoni, A. D., Norman, S. B., and Simmons, A. N. (2020). Neurocomputational changes in inhibitory control associated with prolonged exposure therapy. *Journal of traumatic stress* 33, 500–510

Heinzel, S., Lorenz, R. C., Pelz, P., Heinz, A., Walter, H., Kathmann, N., et al. (2016). Neural correlates of training and transfer effects in working memory in older adults. *Neuroimage* 134, 236–249

Hiroyasu, T., Obuchi, S., Tanaka, M., Okamura, T., and Yamamoto, U. (2015). Working memory training strategies and their influence on changes in brain activity and white matter. In *Proceedings of the 18th Asia Pacific Symposium on Intelligent and Evolutionary Systems-Volume 2* (Springer), 267–278

Hoekzema, E., Carmona, S., Tremols, V., Gispert, J. D., Guitart, M., Fauquet, J., et al. (2010). Enhanced neural activity in frontal and cerebellar circuits after cognitive training in children with attention-deficit/hyperactivity disorder. *Human Brain Mapping* 31, 1942–1950

Hsu, C. L., Best, J. R., Davis, J. C., Nagamatsu, L. S., Wang, S., Boyd, L. A., et al. (2018). Aerobic exercise promotes executive functions and impacts functional neural activity among older adults with vascular cognitive impairment. *British journal of sports medicine* 52, 184–191

Huyser, C., Veltman, D. J., Wolters, L. H., de Haan, E., and Boer, F. (2010). Functional magnetic resonance imaging during planning before and after cognitive-behavioral therapy in pediatric obsessive-compulsive disorder. *Journal of the American Academy of Child & Adolescent Psychiatry* 49, 1238–1248

Huyser, C., Veltman, D. J., Wolters, L. H., de Haan, E., and Boer, F. (2011). Developmental aspects of error and high-conflict-related brain activity in pediatric obsessive–compulsive disorder: a fmri study with a flanker task before and after cbt. *Journal of Child Psychology and Psychiatry* 52, 1251–1260

Hwang, J. Y., Kim, N., Kim, S., Park, J., Choi, J.-W., Kim, S. J., et al. (2019). Stroop task-related brain activity in patients with insomnia: Changes after cognitive-behavioral therapy for insomnia. *Behavioral Sleep Medicine* 17, 621–633

Jolles, D. D., Grol, M. J., Van Buchem, M. A., Rombouts, S. A., and Crone, E. A. (2010). Practice effects in the brain: changes in cerebral activation after working memory practice depend on task demands. *Neuroimage* 52, 658–668

Jolles, D. D., van Buchem, M. A., Rombouts, S. A., and Crone, E. A. (2012). Practice effects in the developing brain: A pilot study. *Developmental Cognitive Neuroscience* 2, S180–S191

Kang, C., Fu, Y., Wu, J., Ma, F., Lu, C., and Guo, T. (2017). Short-term language switching training tunes the neural correlates of cognitive control in bilingual language production. *Human Brain Mapping* 38, 5859–5870

Kelly, C. E., Thompson, D. K., Chen, J., Josev, E. K., Pascoe, L., Spencer-Smith, M. M., et al. (2020). Working memory training and brain structure and function in extremely preterm or extremely low birth weight children. *Human Brain Mapping* 41, 684–696

Kim, H., Chey, J., and Lee, S. (2017). Effects of multicomponent training of cognitive control on cognitive function and brain activation in older adults. *Neuroscience research* 124, 8–15

Krafft, C. E., Schwarz, N. F., Chi, L., Weinberger, A. L., Schaeffer, D. J., Pierce, J. E., et al. (2014). An 8-month randomized controlled exercise trial alters brain activation during cognitive tasks in overweight children. *Obesity* 22, 232–242

Martinsen, S., Flodin, P., Berrebi, J., Löfgren, M., Bileviciute-Ljungar, I., Mannerkorpi, K., et al. (2018). The role of long-term physical exercise on performance and brain activation during the stroop colour word task in fibromyalgia patients. *Clinical physiology and functional imaging* 38, 508–516

Meusel, L.-A. C., Hall, G. B., Fougere, P., McKinnon, M. C., and MacQueen, G. M. (2013). Neural correlates of cognitive remediation in patients with mood disorders. *Psychiatry Research: Neuroimaging* 214, 142–152

Miró-Padilla, A., Bueichekú, E., Ventura-Campos, N., Flores-Compañ, M.-J., Parcet, M. A., and Ávila, C. (2019). Long-term brain effects of n-back training: an fmri study. *Brain Imaging and Behavior* 13, 1115–1127

Nash, K., Stevens, S., Clairman, H., and Rovet, J. (2017). Preliminary findings that a targeted intervention leads to altered brain function in children with fetal alcohol spectrum disorder. *Brain Sciences* 8, 7

Necka, E., Gruszka, A., Hampshire, A., Sarzyńska-Wawer, J., Anicai, A.-E., Orzechowski, J., et al. (2021). The effects of working memory training on brain activity. *Brain Sciences* 11, 155

Newman, S. D., Hansen, M. T., and Gutierrez, A. (2016). An fmri study of the impact of block building and board games on spatial ability. *Frontiers in psychology* 7, 1278

Nishiguchi, S., Yamada, M., Tanigawa, T., Sekiyama, K., Kawagoe, T., Suzuki, M., et al. (2015). A 12-week physical and cognitive exercise program can improve cognitive function and neural efficiency in community-dwelling older adults: a randomized controlled trial. *Journal of the American Geriatrics Society* 63, 1355–1363

Nombela, C., Bustillo, P. J., Castell, P. F., Sanchez, L., Medina, V., and Herrero, M. T. (2011). Cognitive rehabilitation in parkinson's disease: evidence from neuroimaging. *Frontiers in neurology* 2, 82

Olesen, P. J., Westerberg, H., and Klingberg, T. (2004). Increased prefrontal and parietal activity after training of working memory. *Nature neuroscience* 7, 75–79

Osaka, M., Otsuka, Y., and Osaka, N. (2012). Verbal to visual code switching improves working memory in older adults: an fmri study. *Frontiers in human neuroscience* 6, 24

Ott, C. V., Macoveanu, J., Bowie, C. R., Fisher, P. M., Knudsen, G. M., Kessing, L. V., et al. (2021). Change in prefrontal activity and executive functions after action-based cognitive remediation in bipolar disorder: a randomized controlled trial. *Neuropsychopharmacology* 46, 1113–1121

Pensel, M., Daamen, M., Scheef, L., Knigge, H. U., Rojas Vega, S., Martin, J., et al. (2018). Executive control processes are associated with individual fitness outcomes following regular exercise training: blood lactate profile curves and neuroimaging findings. *Scientific reports* 8, 1–12

Ramsay, I. S., Nienow, T. M., and MacDonald III, A. W. (2017). Increases in intrinsic thalamocortical connectivity and overall cognition following cognitive remediation in chronic schizophrenia. *Biological Psychiatry: Cognitive Neuroscience and Neuroimaging* 2, 355–362

Salminen, T., Kühn, S., Frensch, P. A., and Schubert, T. (2016). Transfer after dual n-back training depends on striatal activation change. *Journal of Neuroscience* 36, 10198–10213

Siniatchkin, M., Glatthaar, N., von Müller, G. G., Prehn-Kristensen, A., Wolff, S., Knöchel, S., et al. (2012). Behavioural treatment increases activity in the cognitive neuronal networks in children with attention deficit/hyperactivity disorder. *Brain topography* 25, 332–344

Thorsen, A. L., de Wit, S. J., Hagland, P., Ousdal, O. T., Hansen, B., Hagen, K., et al. (2020). Stable inhibition-related inferior frontal hypoactivation and fronto-limbic hyperconnectivity in obsessive–compulsive disorder after concentrated exposure therapy. *NeuroImage: Clinical* 28, 102460

van der Straten, A., Huyser, C., Wolters, L., Denys, D., and van Wingen, G. (2018). Long-term effects of cognitive behavioral therapy on planning and prefrontal cortex function in pediatric obsessive-compulsive disorder. *Biological Psychiatry: Cognitive Neuroscience and Neuroimaging* 3, 320–328

Wu, M.-T., Tang, P.-F., Goh, J. O., Chou, T.-L., Chang, Y.-K., Hsu, Y.-C., et al. (2018). Task-switching performance improvements after tai chi chuan training are associated with greater prefrontal activation in older adults. *Frontiers in aging neuroscience* 10, 280

Wykes, T., Brammer, M., Mellers, J., Bray, P., Reeder, C., Williams, C., et al. (2002). Effects on the brain of a psychological treatment: cognitive remediation therapy: functional magnetic resonance imaging in schizophrenia. *The British Journal of Psychiatry* 181, 144–152
